# The effectiveness of lixisenatide as an add on therapy to basal insulin in diabetic type 2 patients previously treated with different insulin regimes: a multi-center observational study

**DOI:** 10.1186/s13098-018-0321-x

**Published:** 2018-03-13

**Authors:** Tomislav Božek, Ines Bilić-Ćurčić, Maja Cigrovski Berković, Marina Gradišer, Tina Tićinović Kurir, Sanja Klobučar Majanović, Srećko Marušić

**Affiliations:** 1 University Clinic for Diabetes Vuk Vrhovac, Zagreb, Croatia; 2 Department of Pharmacology, Faculty of Medicine, University J. J. Strossmayer Osijek, Clinical Hospital Center, Osijek, J. Huttlera 4, 31000 Osijek, Croatia; 3 Department for Endocrinology, Diabetes and Metabolism, University Hospital Centre “Sestre Milosrdnice”, Zagreb, Croatia; 4 Internal Medicine Ward, General Hospital Čakovec, Čakovec, Croatia; 5 Department for Endocrinology, Diabetes and Metabolism, University Hospital Center Split, Split, Croatia; 6 Department for Endocrinology, Diabetes and Metabolism, University Hospital Center Rijeka, Rijeka, Croatia; 7 Department for Endocrinology, Diabetes and Metabolism, Clinical Hospital Dubrava, Zagreb, Croatia

**Keywords:** Insulin therapy, Incretin therapies, Novel agents, Obesity

## Abstract

**Introduction:**

This observational study aimed to assess the effectiveness of lixisenatide as add on therapy to basal insulin in diabetic type 2 patients previously treated with different insulin regimes.

**Methods:**

Patients with diabetes type 2, prescribed with lixisenatide and basal insulin were divided in three groups (premixed insulin, basal bolus insulin and basal oral therapy (BOT). Difference in mean change in HbA1c, body mass index, total insulin doses, fasting blood glucose (FPG) and prandial blood glucose (PPG) were assessed after 3–6-months of follow-up.

**Results:**

The primary outcomes were assessed in 111 patients. Lixisenatide added to basal insulin, reduced HbA1c and body weight significantly in all three groups of patients (p < 0.001 for all), with the most prominent reduction in the basal bolus group of patients which had the highest baseline HbA1c compared to premix and BOT treatment groups. Regarding a difference in total insulin dose the reduction was statistically significant in the basal bolus (p = 0.006) and premix group (p < 0.001). FPG and PPG were also significantly reduced over time in all three groups (p < 0.001 for all). A composite outcome (reduction of HbA1c below 7% (53 mmol/mol) with any weight loss) was achieved in 27% of total patients included in the study, reduction of HbA1c below 7% was observed in 30% of patients, while 90% of patients experienced weight reduction.

**Conclusion:**

These results indicate that lixisenatide add on basal insulin treatment (BIT) can improve glycemic control in a population with long-standing type 2 diabetes and previously uncontrolled on other insulin therapy.

## Introduction

Type 2 diabetes is designated by a chronic progressive course and a subsequent need for a long-term insulin therapy to achieve optimal glucose control. Still, a substantial number of T2DM patients do not achieve optimal glucose control despite intensive insulin treatment [1, 2].

Strong rationale exists for the usage of basal insulin in combination with treatments that address postprandial glucose, in order to achieve target HbA1c. In comparison to the previous options, when the basal insulin regime was intensified by adding short-acting insulin, today adding GLP-1 RA to basal insulin represents alternative to adding prandial insulin [3–5].

Although lixisenatide as add on therapy to basal insulin has demonstrated equal efficacy as basal bolus insulin therapy in a head-to-head clinical trial [6], real-world evidence of comparative effectiveness is lacking. The aim of this observational study was to assess the effectiveness of lixisenatide as an add on therapy to basal insulin in diabetic type 2 patients previously treated with different insulin regimes in real world setting.

## Patients and methods

### Patients

This was a multicenter observational study conducted at five tertiary and secondary hospital centers in Croatia (Zagreb, Osijek, Split and Čakovec). Patients were recruited from diabetes outpatient clinics and data from electronic medical records were taken retrospectively and analyzed starting from 2014 until 2017 since lixisenatide has become available in Croatia in May 2014. The study included 111 patients (43 male, 68 female), diagnosed with type 2 diabetes aged 20–80 years, inadequately controlled (HbA1c level ≥ 7 (53 mmol/mol) and ≤ 11% (97 mmol/mol)) on different insulin regimes; premix insulin analogues (45%), basal-bolus regimen (13.5%) or basal supported oral therapy (41.4%). All subjects were GLP 1 RA naïve and consequently prescribed lixisenatide and basal insulin for at least 3–6 months.

This study complied with the Declaration of Helsinki and was approved by the ethics committees. Informed consent was obtained from all patients included in the study.

### Clinical measurements

The following data were collected at baseline and follow up visits (after 3–6 months); age, duration of diabetes, sex, diabetic medications, HbA1c, weight, height, BMI, fasting and postprandial blood glucose levels. Changes in HbA1c, fasting blood glucose, postprandial blood glucose, weight, BMI, were assessed and analyzed. The primary study endpoint was the proportion of participants achieving HbA1c < 7.0% (53 mmol/mol) and/or body weight reduction. Secondary endpoint included changes in insulin doses, FPG and PPG.

### Statistical analysis

Descriptive statistics was used to describe baseline characteristics of the study sample (proportions for categorical data, and mean ± standard deviation for normally distributed continuous variables). Categorical variables of composite outcomes (reduction in weight gain and HbA1c) between three groups according to regimen were analyzed with Chi square test. Two-way repeated-measures ANOVA were used to determine change in given parameters over follow-up period, regarding three groups of patients (with Scheffe’s post hoc test, and Bonferroni correction for multiple comparisons). All statistical comparisons are two-tailed and they were considered significant at the p < 0.05.

## Results

The primary outcome was assessed in 111 patients, 43 (38.7%) males and 68 (61.3%) females.

Subjects’ characteristics at baseline and after follow up period of 3–6 months are presented in Table 1. Average duration of diabetes was 9.6 ± 5.7 years, average age of participants was 62.9 ± 9.4 years and median insulin treatment duration was 20 months. Majority of patients applied basal insulin in the evening 78 (73.7%), while lixisenatide was administered prior breakfast or lunch in equal proportion (43.6% vs. 53.6%).

**Table 1:** Comparison of data obtained at baseline and after 3–6 months of follow up in all three groups of patients

Type of insulin regime (n)	Parameters	Baseline	Control visit	p value
		Mean ± SD	Mean ± SD	
Premix (50)	Weight (kg)	104 ± 14	99 ± 13	0.003
	BMI (kg/m^2^)	38.1 ± 3.1	36.0 ± 3.1	0.039
	Total daily insulin (IU)	53 ± 21	41 ± 12	< 0.001
	Hba1c (%)	8.4 ± 1.2	7.6 ± 0.9	< 0.001
	FBG (mmol/l)	10.1 ± 2.4	7.7 ± 1.8	< 0.001
	PPG (mmol/l)	10.8 ± 2.5	8.7 ± 2	< 0.001
Basal oral (46)	Weight (kg)	109 ± 12	104 ± 11	0.003
	BMI (kg/m^2^)	38.7 ± 3.3	38.6 ± 10	NS
	Total daily insulin (IU)	35 ± 14	37 ± 11	NS
	Hba1c (%)	8.5 ± 0.9	7.6 ± 0.7	< 0.001
	FBG (mmol/l)	8.3 ± 2.4	7.2 ± 1.6	0.009
	PPG (mmol/l)	11.4 ± 2.1	8.4 ± 1.7	< 0.001
Basal bolus (15)	Weight (kg)	107 ± 19	98 ± 14	< 0.001
	BMI (kg/m^2^)	36.0 ± 1	33.8 ± 1.1	0.039
	Total daily insulin (IU)	71 ± 27	50 ± 24	0.006
	Hba1c (%)	9.6 ± 1.8	7.5 ± 0.9	< 0.001
	FBG (mmol/l)	10.3 ± 3	7.6 ± 1.4	< 0.001
	PPG (mmol/l)	12.4 ± 2.9	9.2 ± 2.5	< 0.001

*BMI* body mass index, *FPG* fasting plasma glucose, *PPG* postprandial plasma glucose

In the group of patients previously treated with premix insulins average age of patients was 67.4 years with average duration of diabetes 12.6 years as opposed to basal bolus and basal oral therapy with average age of patients 58.5 and 59.4 years and average duration of diabetes 6.8 and 7.8 years respectively.

**Fig. 1:**
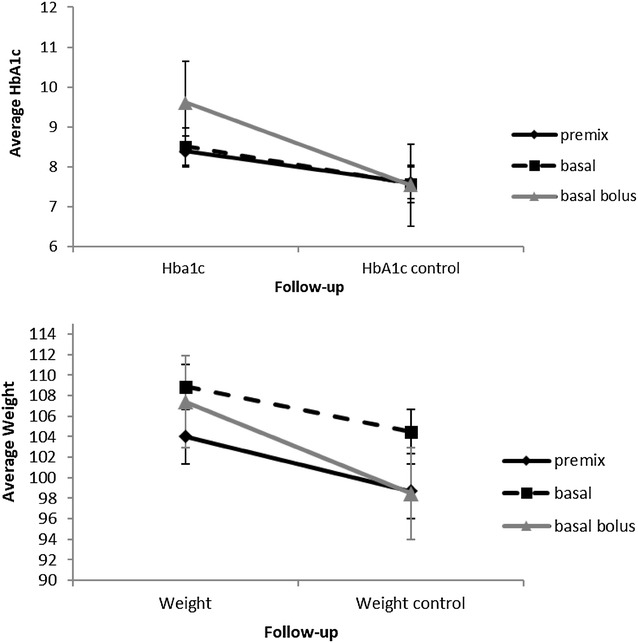
Change of HbA1c and body weight over a follow up period of 3–6 months

Lixisenatide added to basal insulin, reduced HbA1c significantly in all three groups of patients (p < 0.001 for all), with the most prominent reduction in the basal bolus group of patients. (p < 0.001; 2% reduction vs. 0.6 and 0.8% reduction in premix and BOT group respectively) which had the highest baseline HbA1c compared to premix and BOT treatment groups (9.6 ± 1.8% vs. 8.4 ± 1.2 and 8.5 ± 0.9) Fig. 1.

The average body weight of the subjects significantly decreased over time (p < 0.001) in all three groups. The greatest decrease occurred in the basal bolus group with smaller decline in the other two groups (p = 0.003) Fig. 1.

Regarding a difference in total insulin dose the reduction was statistically significant in the basal bolus (p = 0.006) and premix group (p < 0.001), but not in the basal oral therapy group where a slight increase in the average dose of basal insulin was noted. Dose of basal insulin did not change significantly from baseline to follow up visit.

FPG and PPG were also significantly reduced over time in all three groups (p < 0.001 for all). Smallest reduction in FPG occurred in the basal oral therapy group (p = 0.009), while there were no significant differences in PPG reduction between groups.

A composite outcome (reduction of HbA1c below 7% (53 mmol/mol) with any weight loss) was achieved in 27% of total patients included in the study, reduction of HbA1c below 7% (53 mmol/mol) was observed in 30% of patients, while 90% of patients experienced weight reduction (Table 2). Largest proportion of patients achieving composite outcome was in premix group (13.5%) followed by BOT (9%) and basal bolus group (4.5%), If we look at the reduction of HbA1c below 7% (53 mmol/mol) alone then similar distribution among the groups was observed. In addition, decrease of body weight was noted in 90% of patients, majority of them being in premix and BOT groups followed by basal bolus. However, difference for each outcome was not statistically significant among different groups.

**Table 2:** Assesment of outcomes according to groups and total number of patients

Type of insulin regime	Outcome	n	%	p value
Premix	Decrease in both BMI and HbA1c < 7%	15	13.5	
	Decrease in body weight	45	40.9	NS
	Decrease in HbA1c < 7	16	14.5	
Basal	Decrease in both BMI and HbA1c < 7%	10	9	
	Decrease in body weight	39	35.5	NS
	Decrease in HbA1c < 7	13	11.8	
Basal bolus	Decrease in both BMI and HbA1c < 7%	5	4.5	
	Decrease in body weight	15	13.5	NS
	Decrease in HbA1c < 7	5	4.5	

*BMI* body mass index, *NS* not significant

## Discussion

Meta-analysis of 15 studies demonstrated that the combination of GLP-1 RA and basal insulin, in comparison with other anti-diabetic treatments, can enable achievement of robust glycemic control, without increased risk of hypoglycemia and weight gain [7].

Our results are in agreement with GetGoal-L and GG-Duo1 randomized clinical trials (RCT) [8, 9]. In both studies lixisenatide led to a significant decrease in HbA1c up to − 0.7%, lowered body weight and was associated with lower insulin dose. In conclusion, lixisenatide could be considered as an alternative to prandial insulin in T2DM patients sub-optimally controlled on basal insulin which is now supported with real life data.

The GetGoal Duo 2 study is the first trial to directly compare lixisenatide with prandial insulin in combination with basal insulin [6]. The results of the study showed that lixisenatide brings the combined benefit of HbA1c management in line with bolus insulins but with weight reduction as opposed to the usual weight gain and with lower risk of hypoglycemia and lower total insulin dose. Similarly, results of the LIRA-ADD2BASAL study where liraglutide was added to preexisting basal insulin, showed significantly more patients within glycemic targets with addition of this long acting GLP1-RA, and also higher proportion of patients reaching composite end-points, with additional weight reduction and low hypoglycemia risk [10]. Recently published data on DUAL VII RCT showed superior results of fixed combination of IDegLira compared to basal-bolus insulin regimen in risk of hypoglycemia and weight at comparable glycemic control [11], bringing the combination of GLP1-RA and basal insulin in the spot light of treatment intensification of T2DM.

In our real-life study, we have provided evidence of additional benefits besides weight reduction and decrease in total insulin doses such as significant reduction in HbA1c in majority of patients. Furthermore, this combination is interesting also due to the pathophysiological background of DMT2, while beta-cell function is severely impaired in progressed DMT2, and short acting glucagon-like peptide-1 receptor agonists (GLP1-RA) has a pronounced effect on GIT motility [12].

It is evident that insulin initiation is delayed with multiple oral antidiabetic (OAD) combinations, but one of the biggest challenge in clinical practice especially in Croatia are sub-optimally regulated premix and basal bolus patients who often come with the problems of hypoglycemia and weight gain. In addition, health reimbursement restrictions for GLP1-RA in Croatia (BMI > 35) reduces the pool of potential patients for GLP1 therapy [13]. However, according to our data those patients with BMI over 35 kg/m^2^ treated with different insulin regimes regardless of baseline HbA1c level and duration of diabetes could benefit from this specific combination with regard to not only weight and hypoglycemia reduction, but also HbA1c improvement. The biggest success of this change in treatment was noted in patients treated with premix insulins.

The main limitation of this study is non-interventional observational design and availability of only routine data. Also, some clinical events such as hypoglycemic episodes were omitted or underreported which disabled analysis of those data. The strength lays in the uniqueness of data presented since no real life data exist regarding lixisenatide in combination with basal insulin, especially not in previously insulin treated patients.

## Conclusion

Our results indicate and confirm RCT data that lixisenatide add on basal insulin treatment can improve glycemic control in a population with long-standing type 2 diabetes and previously uncontrolled on other insulin therapeutic modalities.
